# Large left ventricular outflow tract mass in a young patient: uncommon presentation of a common disease! A case report

**DOI:** 10.1093/ehjcr/ytae387

**Published:** 2024-09-06

**Authors:** Shivam Goel, Pradeep Ramakrishnan, Sreelal Variar, Sudheer Kumar Arava, Sourabh Agstam

**Affiliations:** Department of Cardiology, All India Institute of Medical Sciences, Room no.11, 7th floor, Cardio-Neuro Centre, AIIMS, New Delhi 110049, India; Department of Cardiothoracic and Vascular Surgery, All India Institute of Medical Sciences, New Delhi, India; Department of Cardioradiology, All India Institute of Medical Sciences, New Delhi, India; Department of Pathology, All India Institute of Medical Sciences, New Delhi, India; Department of Cardiology, All India Institute of Medical Sciences, Room no.11, 7th floor, Cardio-Neuro Centre, AIIMS, New Delhi 110049, India

**Keywords:** Cardiac mass, Cardiac MRI, Infective endocarditis, Left ventricular outflow tract, Mitral aortic intervalvular fibrosa, Case report

## Abstract

**Background:**

Infective endocarditis (IE) predominantly involves the cardiac valves. Timely diagnosis and initiation of therapy significantly reduce morbidity and mortality. Infective endocarditis presenting as a large left ventricular outflow tract (LVOT) mass is an atypical manifestation that provides significant challenges to the treating team.

**Case summary:**

A 19-year-young male presented with exertional shortness of breath, palpitations, and presyncope for 4 months with constitutional symptoms for the last 6 months. Two-dimensional echocardiogram showed a large LVOT mass arising from the mitral aortic intervalvular fibrosa causing dynamic severe aortic valve obstruction, moderate aortic regurgitation, and severe mitral regurgitation. He was managed on lines of IE and received intravenous antibiotics. In view of worsening heart failure and cardiogenic shock, he underwent mass excision, mechanical aortic valve replacement, and mitral valve repair. Histopathology confirmed it as vegetation. He was discharged and is doing well at 2-month follow-up.

**Discussion:**

An atypical presentation of IE as a large LVOT mass was observed in this young male. Sound clinical judgement, judicious use of ancillary imaging, and a multidisciplinary approach ensured timely diagnosis and appropriate treatment. Management included appropriate intravenous antibiotics followed by surgery.

**Learning points**

- Infective endocarditis (IE) is often under recognized and under treated due to its non-specific, atypical, and protean manifestations.
- Infective endocarditis presenting as a LVOT mass with epicentre at mitral aortic intervalvular fibrosa is rare.
- Timely diagnosis and early initiation of antibiotics reduce the mortality in IE.
- Surgery should not be delayed in cardiogenic shock secondary to IE.

## Introduction

Infective endocarditis (IE) is an infection of the endocardial surface of the heart. Despite advances in medical theory, imaging, and practice, it is often under recognized and under treated due to its non-specific, atypical, and protean manifestations. The diagnosis of IE is reached by integrating clinical, microbiological, and imaging findings as described in the 2023 ESC–modified diagnostic criteria for IE.^1^ However, it must be noted that sound clinical judgement cannot be replaced by any diagnostic device and is paramount in guiding evaluation and management in atypical presentations. Herein, we present the case of a young adult presenting with congestive heart failure due to a large left ventricular outflow tract (LVOT) mass which was confirmed as IE following surgical excision.

## Summary figure

**Table:** 

6 months prior to admission	Insidious onset loss of appetite, loss of weight, and low-grade fever
4 months prior to admission	Exertional presyncope, shortness of breath, and palpitations
3 months prior to admission	Exertional syncope
Day 0 (in hospital)	Patient was in congestive heart failure. Two-dimensional transthoracic echocardiogram showed large LVOT mass arising from the mitral aortic intervalvular fibrosa (MAIF) and prolapsing into the aortic valve causing dynamic obstruction and moderate aortic regurgitation. Restriction of anterior mitral leaflet causing severe eccentric mitral regurgitation. Patient was managed on lines of possible IE and started on empirical vancomycin and gentamicin
Day 3	3 sets of blood cultures drawn prior to antibiotic administration reported as sterile
Day 5	On cardiac magnetic resonance imaging (MRI), tissue characterization of mass suggestive of thrombus or vegetation ruled out neoplasm
Day 12–17	Worsening heart failure with development of cardiogenic shock requiring inotropic support
Day 17–20	Surgical excision of LVOT mass, mechanical aortic valve replacement, and mitral valve repair. Histopathology was suggestive of vegetation, and tissue culture was sterile
Day 53	Completion of course of intravenous antibiotics for 6 weeks. The patient was discharged and doing well in the follow-up

## Case presentation

A 19-year-young male presented to the emergency department of our tertiary care hospital with complaints of gradually worsening shortness of breath and palpitations for 4 months. He also complained of exertional dizziness and had one episode of exertional syncope 3 months prior to presentation. On direct questioning, he reported low-grade fever, significant unintentional weight loss, and loss of appetite for 6 months.

At presentation, the blood pressure was 88/52 mmHg in right upper limb and 125/45 mmHg in right lower limb with a resting heart rate of 120 b.p.m. The pulse was regular, normal volume with no special character. He was tachypnoeic (respiratory rate of 26 breaths per minute) on room air with saturation of 98%. The jugular venous pressure was raised, and grade 2 generalized clubbing was noticed. There was no evidence of embolic or immunological phenomenon of IE and no signs of acute rheumatic fever. On auscultation, a late peaking grade 3/6 ejection systolic murmur and a high pitched early diastolic murmur were appreciated in left third intercostal space. A grade 3/6 pansystolic murmur radiating to axilla was noted at apex. Bilateral basal fine crepitations were appreciated.

Laboratory investigations showed moderate normocytic, normochromic anaemia (haemoglobin: 9.4 g/dL; normal range: 13–14 g/dL) with elevated reactive C-protein 26 mg/dL (normal range: <0.5 mg/dL), and erythrocyte sedimentation rate of 50 mm/h (normal range: ≤15 mm/h). Blood cultures drawn prior to antibiotic therapy were sterile after standard and prolonged incubation for typical, atypical, and fungal microorganisms. Evaluation for blood culture–negative IE and non-infectious endocarditis was unremarkable.

**Figure 1:**
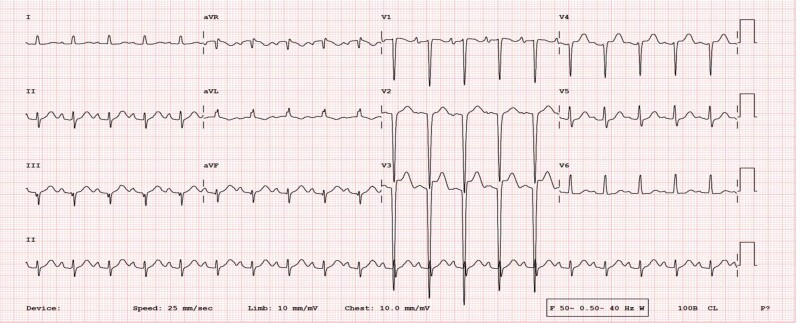
Electrocardiogram at presentation showing sinus tachycardia with left axis deviation and poor R wave progression.

The 12-lead electrocardiogram showed sinus tachycardia, left axis deviation (−60°), poor R wave progression, and deep S wave in early precordial leads suggestive of left ventricular enlargement (*Figure 1*).

**Figure 2:**
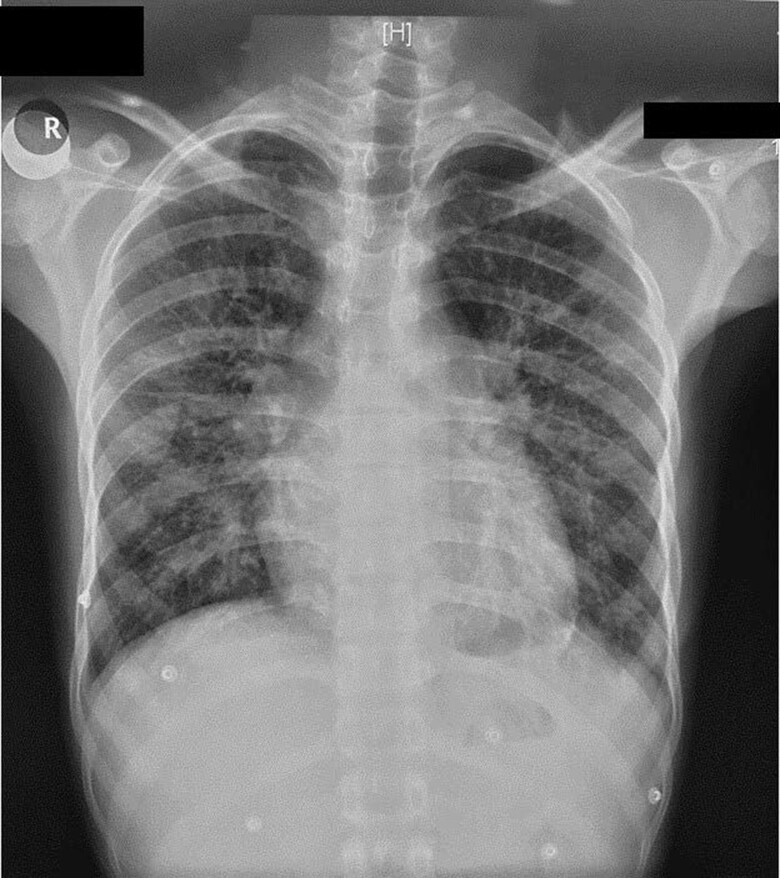
Chest radiograph showing pulmonary venous hypertension.

Chest radiograph showed a cardiothoracic ratio of 0.45, cephalization of pulmonary veins, and fissural oedema suggestive of pulmonary venous hypertension (*Figure 2*).

Transthoracic echocardiogram showed a large mass arising from the MAIF, prolapsing into the LVOT during systole, and impinging on non-coronary cusp of aortic valve (see Supplementary material online, *Videos S1* and *S2*). This caused dynamic aortic valve obstruction and moderate eccentric aortic regurgitation with jet directed towards the anterior mitral leaflet (see Supplementary material online, *Video S1*). The anterior mitral leaflet had limited systolic excursion leading to severe eccentric mitral regurgitation with posteriorly directed jet (see Supplementary material online, *Video S1*). Mild tricuspid regurgitation and severe pulmonary hypertension were present.

Considering the clinical presentation, epidemiology, and location of the mass, a differential diagnosis of IE as large vegetation, a LVOT myxoma, or an infected cardiac tumour was made. A possible diagnosis of IE was established by the 2023 ESC–modified diagnostic criteria (one major plus one minor criterion). In view of community acquired native valve IE, following consultation with our multidisciplinary heart team, consideration of local epidemiology, and institutional antibiotic stewardship policy, he was started empirically on intravenous antibiotic therapy with vancomycin and gentamicin and low dose of intravenous diuretics for heart failure.

Considering the implications for management, it was decided to pursue further imaging to better characterize the lesion, delineate the relevant anatomy, and resolve the diagnostic dilemma.

**Figure 3:**
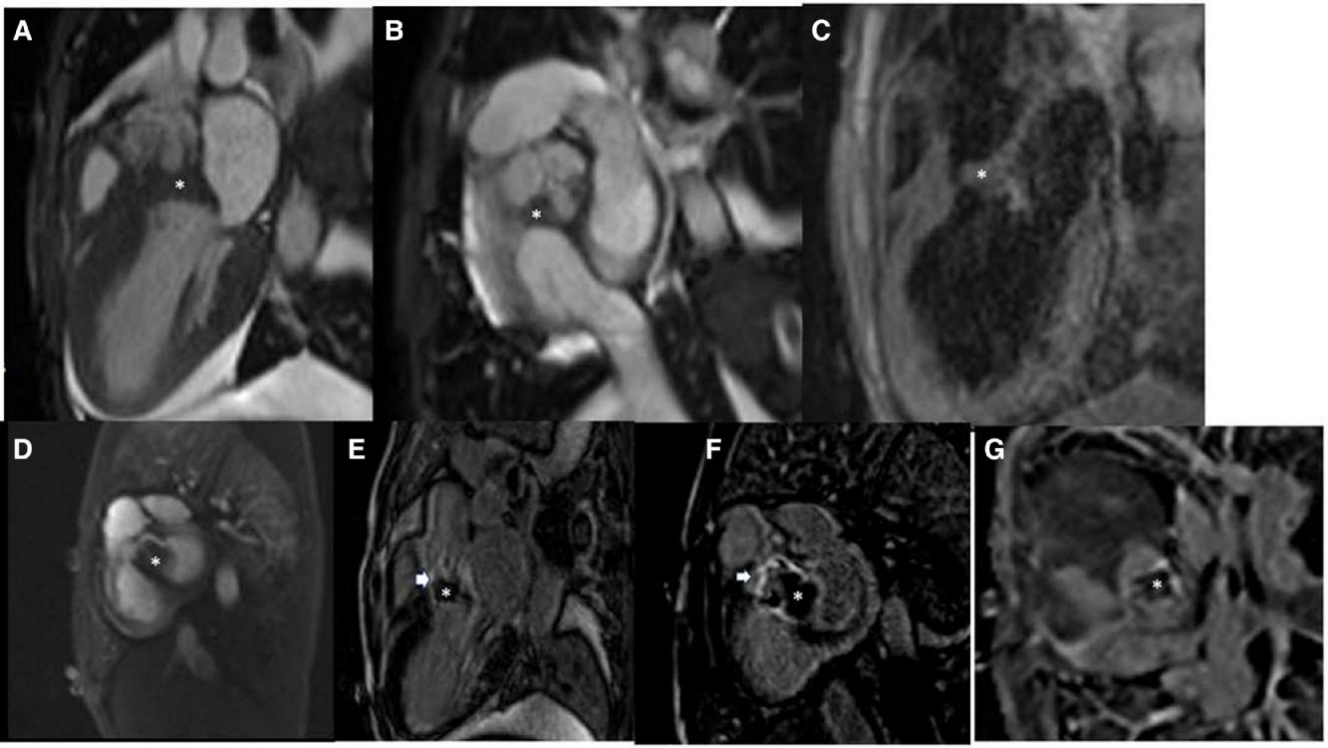
Cardiac MRI. (*A*)–(*G*) showing left ventricular outflow tract mass (*). (*A*) (left ventricular outflow tract view) and (*B*) (short-axis view) showing mass in the left ventricular outflow tract with aortic valve involvement. (*C*) (T2 dark blood sequence) showing isointense nature of the mass with myocardium. (*D*) (first-pass perfusion) showing no enhancement of the mass. (*E*) and (*F*) showing peripheral enhancement of the mass (white arrows). (*G*) is high TI (800 ms) sequence showing hypointense central core of the mass, suggestive of thrombus.

Cardiac magnetic resonance imaging (MRI) (cardiovascular magnetic resonance [CMR]) showed a 12 × 22 × 18 mm lesion with the epicentre at the MAIF, with associated thickening of anterior mitral leaflet and distortion of non-coronary and right coronary cusp of aortic valve with eccentric calcifications. It was hyperintense on T1 weighted sequences, iso-hyperintense on T2 weighted and T2 fat-sat sequences. There was no evidence of perfusion on dynamic perfusion imaging. Heterogeneous late gadolinium enhancement was seen at the periphery of the lesion, LVOT, paravalvular region of aortic valve and anterior mitral valve. Central area of the lesion appeared hypointense on high T1 sequence. These findings were consistent with thrombotic nature of the lesion (*Figure 3*).

Considering the likely infective aetiology and clinical profile, treatment options were discussed with the heart team and the patient, including the benefit of antibiotic therapy prior to surgical excision vs. an early surgery performed upfront. The consensus was that the risk of prosthetic valve endocarditis outweighed the benefits provided by early surgery for the patient. Accordingly, he was admitted under intensive care and continued intravenous antibiotics.

During the hospital stay, he developed progressively worsening shortness of breath (NYHA IV) and orthopnoea. He had cold extremities, increased bilateral fine crepitations, and decreased urine output. The blood pressure reduced to 70/40 mmHg. Laboratory investigations revealed increased serum lactate and acute kidney injury (AKIN II). He was started on intravenous inotropic support and planned for urgent surgery.

**Figure 4:**
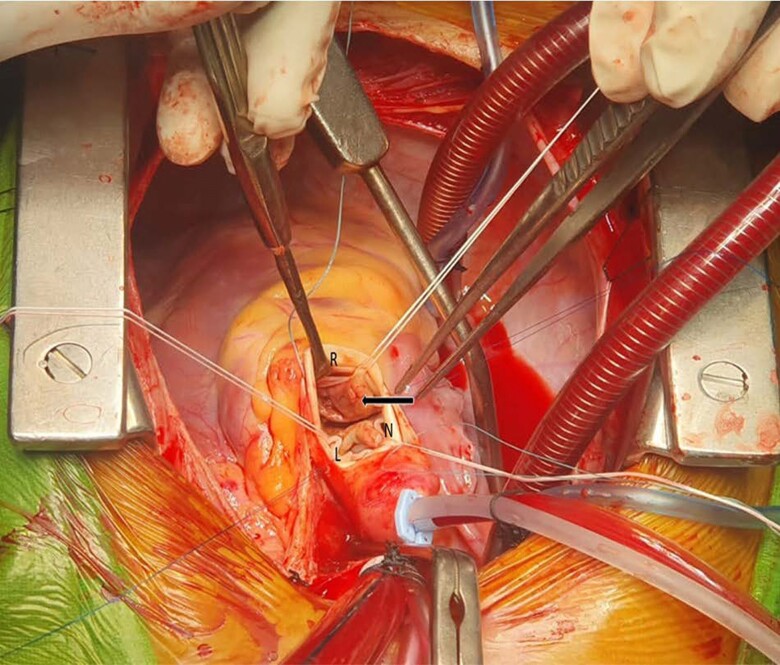
Intraoperative appearance of left ventricular outflow tract mass (transaortic view): intraoperative transaortic view of the mass with near complete obstruction of the left ventricular outflow tract (arrow) with destroyed non-coronary (N) and left coronary (L) aortic valve cusps and intact right coronary (R) cusp.

**Figure 5:**
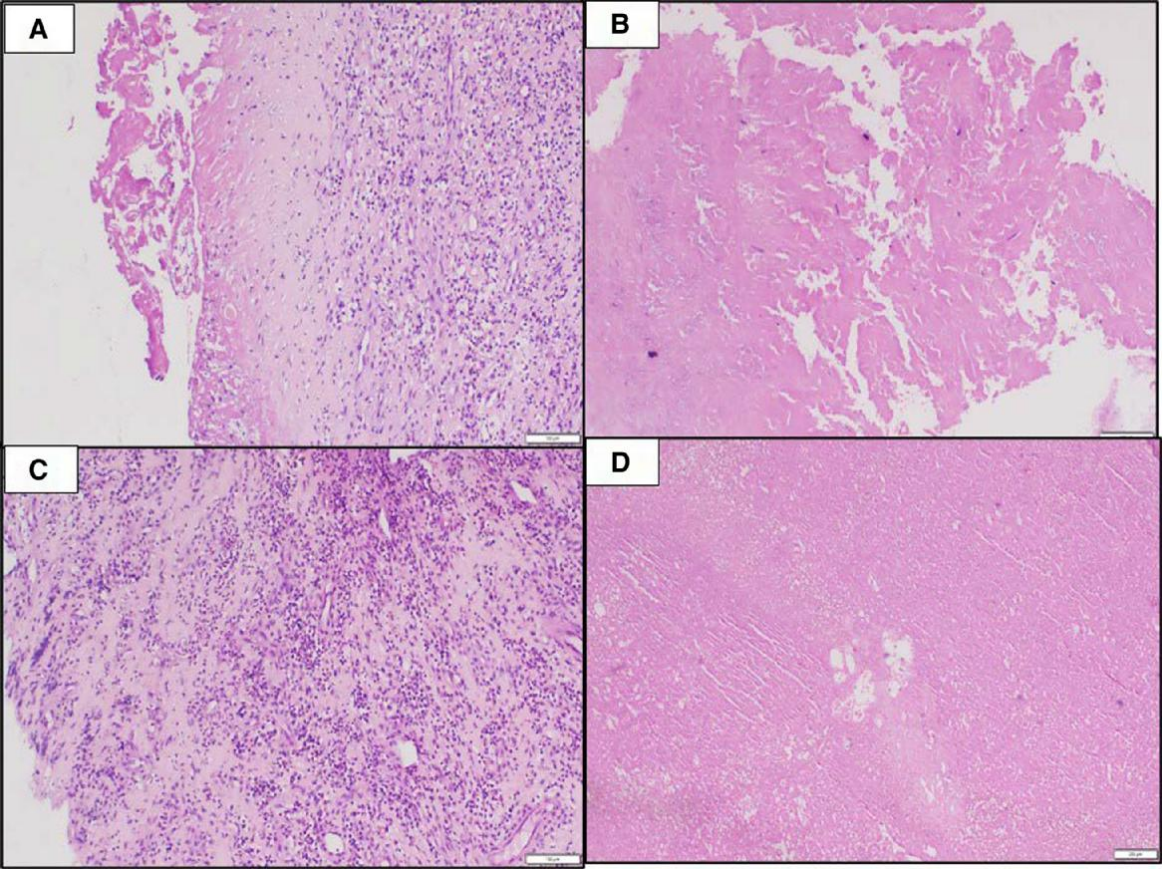
Histopathology of excised aortic valve: fibrosis, neovascularization (*A*) with large fibrin-rich vegetation (*B*) and neutrophilic debris (*C*) and excised left ventricular outflow tract mass (*D*): fibrin-rich vegetation with focal organization and neutrophilic debris.

On Day 17 of admission, he underwent transaortic mass excision, aortic valve replacement with bi-leaflet mechanical prosthetic valve (18 mm, Miltonia, Merillife), and mitral valve repair (*Figure 4*). Intraoperatively, the non-coronary cusp was found to be destroyed and left coronary cusp as perforated. Histopathology of the mass was suggestive of vegetation with inflammation of the underlying aortic valve (*Figure 5*). No organism was identified on routine microbiological stains. Aortic valve and excised mass cultures were sterile.

Since an aetiological microorganism could not be isolated, it was decided in consultation with the heart team to continue intravenous antibiotics (vancomycin and gentamicin) for 6 weeks. On Day 53 of hospitalization, he was discharged with advice for close outpatient follow-up. In follow-up consultations at 2 weeks and 6 weeks, he continues to be asymptomatic with preserved valve function and no new adverse clinical events.

## Discussion

We described the case of a young man with a large LVOT mass at the MAIF presenting as a diagnostic dilemma. The non-specific initial symptoms, absence of known risk factors for IE, sterile blood cultures, and no embolic/immunological phenomena made the diagnosis challenging.

The MAIF is an avascular fibrous membrane between the anterior mitral leaflet and aortic valve (non-coronary and left coronary cusp).^2^ It is involved in 40–80% of IE cases following peri-annular extension of infection leading to abscess and pseudoaneurysm formation with complications ranging from compressive effects to fistulization into adjacent structures.^3,4^ Till date, IE presenting as an isolated large vegetation at the MAIF has not been reported.

Concomitant involvement of the aortic and mitral valves in IE is seen in 10–15% of cases.^5^ It is believed to be due to direct proliferation of the infective process from the aortic to mitral valve, impingement of regurgitant aortic jet onto the anterior mitral leaflet or concomitant involvement of both valves.^5^

In our case, based on the patient profile, clinical presentation, and imaging features, we were able to reasonably exclude a neoplasm particularly myxoma. Myxomas are the most common primary cardiac neoplasms which frequently arise from the left atrium, with only 0.7–3.6% of cases having an origin in the left ventricle.^6^ LVOT origin of myxoma is exceedingly rare.^7^ It is difficult to differentiate from an IE vegetation due to similar presentation of constitutional symptoms, embolic phenomenon, and morphological resemblance in atypical cases.^7^ There is also a risk of recurrence following surgical excision. Moreover, in 4–5% of cases of cardiac tumours, there may be seeding and infection of the mass following bacteraemia leading to similar manifestations as IE.^8^

In our case, CMR-enabled non-invasive tissue characterization reasonably ruled out a neoplastic aetiology. There is a growing role of CMR beyond detecting embolic events in IE as mentioned in the current ESC guidelines.^1^

Successful treatment of IE requires completion of a prolonged course of intravenous antibiotics (based on culture and sensitivity patterns). Surgery in the same admission may be contemplated in case of development of heart failure, uncontrolled infection, or embolic events.^1^

## Lead author biography


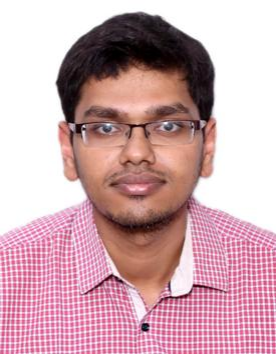


He is a cardiology resident in training at All India Institute of Medical Sciences, New Delhi. He completed his post-graduation in medicine at All India Institute of Medical Sciences, New Delhi. His fields of interest include cardiovascular physiology, heart failure, and congenital heart disease.

## Supplementary Material

ytae387_Supplementary_Data

## Data Availability

The data underlying this article are available in the article and on request to the corresponding author.

## Supplementary material

**Consent:** The authors confirm that written consent for submission and publication of this case report including the images and associated text have been obtained from the patient in line with the COPE guidelines.

**Funding:** None declared.
